# Advances and Opportunities in Nanoparticle Drug Delivery for Central Nervous System Disorders: A Review of Current Advances

**DOI:** 10.7759/cureus.44302

**Published:** 2023-08-29

**Authors:** Chukwuyem Ekhator, Muhammad Qasim Qureshi, Abdul Wasay Zuberi, Muqaddas Hussain, Niraj Sangroula, Sushanth Yerra, Monika Devi, Muhammad Arsal Naseem, Sophia B Bellegarde, Praful R Pendyala

**Affiliations:** 1 Neuro-Oncology, New York Institute of Technology, College of Osteopathic Medicine, Old Westbury, USA; 2 Medicine and Surgery, Mayo Hospital, Lahore, PAK; 3 Medicine and Surgery, Dow University of Health Sciences, Karachi, PAK; 4 Internal Medicine, Mayo Hospital, Lahore, PAK; 5 Psychiatry, College of Medical Scieces, Bharatpur, NPL; 6 Internal Medicine, University of Medicine and Health Sciences, Basseterre, KNA; 7 Medicine, Ziauddin University, Karachi, PAK; 8 Medicine, Mayo Hospital, Lahore, PAK; 9 Pathology and Laboratory Medicine, American University of Antigua, St. John's, ATG; 10 Neurology, Chalmeda Anand Rao Institute of Medical Sciences, Karimnagar, IND

**Keywords:** targeted therapy, cns disorders, nanotechnology, nanoparticle, neurology

## Abstract

This narrative review provides an overview of the current advances, challenges, and opportunities in nanoparticle drug delivery for central nervous system (CNS) disorders. The treatment of central nervous system disorders is challenging due to the blood-brain barrier (BBB), which limits the delivery of therapeutic agents to the brain. Promising approaches to address these issues and improve the efficacy of CNS disease therapies are provided by nanoparticle-based drug delivery systems. Nanoparticles, such as liposomes, polymeric nanoparticles, dendrimers, and solid lipid nanoparticles, can be modified to enhance targeting, stability, and drug-release patterns. They allow for the encapsulation of a variety of therapeutic compounds and can be functionalized with ligands or antibodies for active targeting, minimizing off-target effects. Additionally, nanoparticles can circumvent drug resistance processes and provide versatile platforms for applications that combine therapeutic and diagnostic functions. Although the delivery of CNS medications using nanoparticles has advanced significantly, there are still challenges to be resolved. These include understanding the BBB interactions, doing long-term safety studies, and scaling up the production. However, improvements in nanotechnology and a deeper comprehension of CNS disorders provide opportunities to enhance treatment results and address unmet medical requirements. Future research and ongoing clinical trials are required to further explore the potential of nanoparticle drug delivery for CNS disorders.

## Introduction and background

The treatment of central nervous system (CNS) disorders poses significant challenges due to the blood-brain barrier (BBB), which restricts the passage of most therapeutic agents from systemic circulation to the brain. Over the years, nanoparticle (NP) -based drug delivery systems have emerged as promising solutions to overcome these challenges and improve the efficacy of CNS disorder treatments. Nanoparticles offer unique advantages, such as enhanced drug stability, prolonged circulation time, and the ability to encapsulate a wide range of therapeutic agents. This narrative review provides an overview of the current advances, challenges, and opportunities in nanoparticle drug delivery for CNS disorders.

The BBB functions as a selective barrier, preventing noxious substances from entering the brain while controlling the passage of necessary nutrients and drugs. The delivery of therapeutic medications is nevertheless restricted by its tight junctions and efflux transporters. Drugs can be delivered to the brain effectively by using nanoparticles that can be created to avoid or take advantage of these obstacles [1]. For CNS medication delivery, several formulations based on nanoparticles have been created, including liposomes, polymeric nanoparticles, dendrimers, and solid lipid nanoparticles. These formulations may be modified to increase targeting to certain brain areas, improve stability, and optimize drug-release patterns [2].

Nanoparticles offer the ability to encapsulate a wide range of therapeutic agents, including small molecules, proteins, nucleic acids, and even gene-editing tools. This adaptability makes it possible to give various medications in a single formulation, use combination therapy, and employ personalized medicine techniques [3]. Additionally, ligands or antibodies that recognize receptors overexpressed in sick brain areas can be used to functionalize nanoparticles. While minimizing off-target effects, this active targeting strategy enhances drug accumulation at the target location [4].

Nanoparticles can also circumvent multidrug resistance mechanisms in CNS disorders by modulating drug release kinetics, co-delivering efflux pump inhibitors, or utilizing alternative drug delivery routes to bypass resistant cells [5]. They have the potential to operate as multifunctional platforms that combine medicinal drugs with imaging agents and diagnostic technologies. Real-time monitoring of medication distribution, evaluation of treatment efficacy, and early disease progression detection are all made possible by this capacity. 

Nanoparticle-based drug delivery systems have shown promise for various CNS disorders. Future therapeutic uses for these technologies are possible, as shown by ongoing clinical trials testing their effectiveness and safety. Although nanoparticle-based CNS medication delivery has made great progress, there are still several obstacles to overcome. For nanoparticle formulations to be successfully translated into clinical practice, further research is required to understand how the BBB interacts with them, conduct long-term safety analyses, and scale up manufacturing. However, the ongoing advancements in nanotechnology and the growing understanding of CNS disorders offer tremendous opportunities for improving therapeutic outcomes and addressing unmet medical needs. 

## Review

Opportunities in NP drug delivery for CNS disorders

Enhanced Permeation and Retention Effect

NP-based drugs are helpful, especially because of their ability to permeate the BBB to a greater extent as compared to traditional strategies. Their ability to cross the BBB depends upon their size, shape, surface chemistry, and stability. NPs getting trapped in selected sites like tumors constitute a passively targeted drug delivery system. This phenomenon is named the enhanced permeation and retention effect (EPR effect) [6]. The reason for its occurrence is the presence of leaky vessels due to rapid, defective angiogenesis and poor lymphatic drainage in the affected regions, like tumors. Nanostructures enter the site by either convection within fluids or passive diffusion. The type of NPs that most commonly employ passive targeting drug delivery are the liposomes that can cross the BBB.

Active Targeting Strategies

If we want to achieve maximum efficacy from NPs with limited side effects, we must use techniques to specifically target the cells of the brain requiring treatment. Both non-invasive and invasive techniques have been postulated. Non-invasive biologic methods like antibody-directed, receptor-directed delivery and chemical methods like using a drug conjugate are possible solutions to employ targeted approaches. Invasive ways of targeting can include intracerebral implants [7]. Currently, the most widely used targeting method is attaching a ligand to the surface of an NP and allowing the ligand to interact with its particular receptors, sometimes within a tumor microenvironment [8]. Hence, the ligand guides the NP to its site for specific drug delivery and limiting off-target side effects. Similarly, monoclonal antibodies can be attached to the NP surface to act as a guide. Active targeting, in short, relies on the differences in receptors and antigen expression between normal cells and the target cells. Cytokine-mediated active targeting has been tested in some studies. It was observed that interleukin-13 (IL-13) loaded liposomes used against glioblastoma that had overexpression of IL-13 alpha2 receptors, resulted in a 5-fold reduction of tumor size as compared to free, non-targeted liposomes [9]. Aptamer-mediated and stem cell-mediated active targeting are some of the latest active targeting drug delivery systems. 

Responsive Drug Release

One way to achieve better efficacy in drug delivery with minimal side effects is to practice stimulus-responsive drug release. It means the NPs loaded with the drug will deliver it to the site that provides it with a specific stimulus. The trigger for drug release can be physical, chemical, or biological. Various stimuli like a particular pH at a site, temperature, enzyme activity, redox potential, and light, etc. have been explored. It is well known that tumors create a microenvironment different from the physiologic state. They may exhibit different pH, oxygenation, perfusion, and metabolic state. We can utilize the change of pH in the tumor microenvironment by formulating NPs using a pH-sensitive phospholipid component. Phosphatidylethanolamine (PE) or oleic acid (OA) are widely used substances for conferring pH sensitivity to liposome NPs [10]. Alternatively, we can achieve site-selective drug release by identifying a particular enzyme expressed to a greater extent in a region and developing an enzyme-specific substrate to be acted upon by that enzyme. Among hydrolases, matrix metalloproteinases (MMPs) are of great interest. Several MMP-responsive NPs have been useful in therapeutics and diagnostics [11]. 

Multimodal Imaging and Therapeutic Agents

NPs with combined imaging and therapeutic properties are of special interest to scientists these days. Particularly, magnetic NPs (MNPs) are being extensively explored to this end. A study has demonstrated positive results in using MNPs with an iron-cobalt core and a graphite carbon shell (FeCo/C), delivering small interfering RNA (siRNA) to tumor cells [12]. In addition, the hypothesis that MNPs can work as highly sensitive magnetic resonance and Raman imaging probes was also tested [12]. These multifunctional NPs will be of utmost interest in neurological cancer diagnostics.

Personalized Medicine Approaches

Nanotechnology is paving the way for making personalized medicine a possibility soon. The idea is to use NPs to detect the tumor biomarkers, responsible for the pathogenesis or clinical outcomes in a specific patient, and simultaneously load them with some drug (i.e. using siRNA) which will alter the expression of those tumor biomarkers [13].

NP-based drug delivery systems for CNS disorders

**Figure 1:**
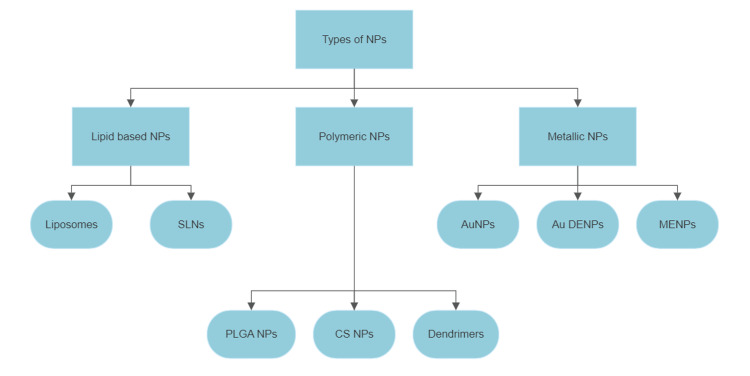
NP-based drug delivery systems for CNS disorders NPs: nanoparticles, SLNs: solid lipid NPs, PLGA NPs: poly (lactic-co-glycolic acid) NPs, CS NPs: chitosan NPs, AuNPs: gold NPs, Au DENPs: dendrimer-entrapped gold NPs, MENPs: magneto-electric NPs.

Nanoparticle-mediated drug delivery systems for CNS disorders encompass a range of formulations, such as lipid-based, polymeric, and metallic nanoparticles (Figure 1).

Lipid-Based NPs 

Liposomes, solid lipid NPs, nanostructured lipid carriers, and nano-emulsions are some of the types of lipid-based NPs [14-16]. They are of great interest in the field of drug delivery because they form lamellar vesicles that resemble the natural membranes of cells. This enables them to be readily taken up by the brain.

Liposomes can vary in size and form unilamellar or multilamellar structures. Their membranes are made of two layers of phospholipids and they can be loaded with drugs [17]. Their short half-life, which was once considered a drawback, has been prolonged by altering their surface using polyethylene glycol (PEG) derivatives. Liposomes are especially helpful in the delivery of large pieces of DNA in a protected space, but the phospholipids can undergo hydrolysis and oxidation hence impairing their function as a carrier [18]. Conventional liposomes were recognized by the reticuloendothelial system of the liver and spleen and hence had a limited half-life. Stealth properties were introduced in liposomes using substances like PEG that minimize the binding of opsonins through steric stabilization [19].

Solid Lipid NPs (SLNs) are solid core lipid nanostructures capable of carrying both hydrophilic and lipophilic drugs [20]. They contain solid lipids that are dispersed in an aqueous solution and also have a surfactant added to them. SLNs have the advantage of offering physical stability and are safe carriers for environment-sensitive labile drugs [21].

SLNs have some drawbacks like drug expulsion during storage, insufficient carrier loading, and large water content. To overcome these, nanostructured lipid carriers (NLCs) were introduced which are a modified version of SLNs. While SLNs have only a solid core, NLCs comprise both solid and liquid lipids [20].

Polymeric NPs

Polymer-based NPs offer some beneficial characteristics that make them a good candidate for an efficient drug delivery system. They have low toxicity, are biocompatible, and enable regulated drug release [22]. Among polymeric NPs, Poly (lactic-co-glycolic acid) (PLGA) is the most effective biocompatible and biodegradable substance used. It has all the properties of polymeric NPs and has received FDA approval [22]. Research is being done to further modify these carriers for further benefit. Study with PLGA NPs has shown that coating them with an RBC membrane resulted in better permeability and higher binding affinity to the target cells than the naked NPs [23]. Other polymeric substances that can be used are polyethylene glycol, polylactic acid, and poly (glutamic acid) among others.

Chitosan (CS), an alkaline polysaccharide derived from chitin, has been used in nanotechnology due to its biocompatibility and easy availability. The presence of active hydroxyl and amino groups in chitosan allows us to introduce different functional groups and modify them according to our requirements. It is believed that chitosan also has some bacteriostatic, fungistatic, spermicidal, and anti-carcinogenic properties. CS NPs have some limitations like their low solubility at neutral and alkaline pH i.e. physiologic pH. This causes low bioavailability due to pre-systemic metabolism after oral intake [24]. 

Dendrimers are three-dimensional polymeric nanostructures with radially symmetric molecules having tree-like branches. The most commonly used dendrimers are the ones derived from polyamidoamine. Their surface can be modified to have various functional groups like hydroxyl, amino, or acid groups. These groups can be conjugated with targeting ligands like Folic acid, allowing us to produce intelligent NPs against specific tumor sites [25]. Dendrimer-conjugated drugs have the advantage of lengthened half-life, greater stability, and decreased immunogenicity or antigenicity [26].

Metallic NPs

Gold NPs (GNPs or AuNPs) can carry small drugs or large molecules like DNA or proteins. Their size can be tuned according to the payload features. These NPs have the advantage that the gold core is nontoxic. GNPs have an added benefit due to their interaction with thiol groups, which allows for controlled intracellular release [27]. Dendrimer-entrapped gold NPs (Au DENPs) are being utilized for computed tomography (CT) imaging applications. This is based on the fact that the dendrimer-stabilized AuNPs display a better x-ray attenuation property and have a longer blood circulation time than conventional iodinated contrast agents [28]. Dendrimer coating also provides colloidal stabilization to the nanostructure. To achieve targeted tumor CT imaging, Folic Acid-linked Au DENPs have been used to target lung adenocarcinoma with overexpression of folic acid receptors. 

Magneto-electric NPs (MENPs) are the latest advancement in nanomedicine with promising future applications. MENPs can work as a two-way interface at the molecular level [29]. They can produce a magneto-electric effect that can act to either stimulate or receive electric signals from their surroundings [30]. This way, we can wirelessly connect with the intracellular process of neurons. The human body, its cells, and its fluids are replete with ions and the membranes are electrically polarized too. All these interacting together create a stable energy state. Previously, physically implanted electrodes have been used to correct any signature alterations in the electric fields inside the brain like in this study for obsessive compulsive disorder (OCD) using deep-brain stimulation (DBS) [31]. MENPs are wireless devices that can overcome the lack of spatial precision which is often a drawback in using neuroelectrodes [29]. 

Application of NP drug delivery for CNS disorders

Alzheimer’s Disease

Conventional orally administered drugs used in neurodegenerative diseases have to pass through first-pass metabolism, crossing BBB and sustainable circulation in the bloodstream. Characteristics of drugs like polarity, molecular weight, net charge, solubility, and affinity for hydro or lipid moieties are mainly responsible for therapeutic failure. The NP drug delivery system modulates these properties and enhances the therapeutic potential of delivered drugs through this system. 

Amyloid [Aβ] deposition is a major pathophysiologic factor in the severity of Alzheimer’s disease. A study confirmed that when functionalized liposomes conjugated with phosphatidic acid and cardiolipin they could effectively target these amyloids with the highest affinity [32]. Another therapeutic approach to treat Alzheimer’s disease is the inhibition of acetylcholine activity which is enhanced in this disease. Rivastigmine, a well-known acetylcholine inhibitor, was experimented with in a preclinical tryout, and as found that its inhibitory activity was enhanced when conjugated with sodium taurochlate liposomal carrier in comparison to its free form [33].

Parkinson’s Disease

Nanotechnology offers a promising future for the treatment of Parkinson’s disease. Dopamine-loaded liposomes were directly implanted in the striatum of a rat and it was found that the levels of dopamine remained elevated for almost 25 days with 40 days of continuous dopamine release after the implantation [34]. Other studies also confirmed the sustained release of levodopa formulations delivered through nanotechnology peripherally [35,36]. Moreover, the ability of NPs to encapsulate the drug offers protection against biodegradation thus increasing their availability to CNS. The toxicity to which a patient suffering from Parkinson’s is exposed is reduced by using cerium oxide NPs. It was found that this NP apart from drug delivery also reduces the dose-dependent toxic effects of alpha-synuclein and it also prevents the oxidative stress caused by alpha-synuclein [37]. 

Multiple Sclerosis

The use of nanotechnology for the treatment of multiple sclerosis is based on its ability to target immune cells and create immune tolerance. NPs can also encapsulate some disease-specific auto antigens and present them to antigen-presenting cells in a way that the resultant immune response is tolerable by the patient [38-40]. This ability of NPs was tested using gold NPs and was found to lead to decreased production of cytokines over time [41]. Nanotechnology favors the direct delivery of drugs to immune cells due to which less amount of drug is required to create a similar effect in comparison to previous therapies. Furthermore, unwanted interactions with non-targeted cells can be avoided thus reducing the side effects of the drugs. Another trial that formed interferon beta NPs concluded that interferon delivered through these NPs was released slowly and a sustained amount was released throughout [42]. This study also found that the impact on cell viability of splenocytes in mice was reduced by increasing the concentration of interferon NPs whereas in low concentrations the effect on cell viability of these NPs was almost similar to free interferon beta treatment. In conclusion, this preclinical trial concluded intranasal administration of interferon beta-loaded NPs as a noninvasive cost-effective therapy for multiple sclerosis with a promising future [42]. 

Brain Tumors

Small interfering RNA and micro RNA are two examples of naked nucleic acids that have the potential to treat gliomas very effectively but their use is limited by their fast degradation, poor internalization, and lack of specificity. NPs were found to prevent this fast degradation and promise a future use of these naked RNA molecules in the treatment of gliomas [43]. NPs are found to be more effective than regular therapies for drug administration in having enhanced penetration, accumulation, distribution, and retention of drugs in glioblastoma cells [44]. Another approach for drug delivery to tumors is using magnetic NPs. These NPs are capable of reacting with certain magnetic fields that enable them to be therapeutic along with diagnostic in brain tumor management [45,46]. Along with targeted drug delivery these magnetic NPs are capable of increasing the temperature within tumor cells and preventing their growth and infiltration [47,48]. 

Traumatic Brain Injury and Stroke 

The altered pH in a stroke can be used by stromal cell-derived factor-1 alpha-loaded, pH-sensitive polymeric micelles to create a favorable microenvironment for neuronal recovery [49]. Targeted delivery of thrombin was also experimented with in a study done by Guo et al. by using conjugating thrombin with lipid NPs in the ischemic brain [50]. To protect neurons from further damage Wang et al. developed a near-infrared light-driven nano-photosynthesis biosystem that had a combination of biological and biochemical systems [51]. Furthermore, another study attempted to incorporate tpA into magnetic microrods for clot dissolution [52]. 

Preclinical studies 

A preclinical tryout was conducted in India on nano lipid carriers containing curcumin delivery into CNS by the intranasal route. The objectives of the study were to enumerate its cytotoxic effects and assess its efficacy. The NP curcumin-loaded nano lipid carriers were produced in this study by hot high-pressure homogenization technique. A surfactant and stabilizer molecule were also used which were Tween 80 and lecithin, respectively. Curcumin was released in a biphasic pattern that was a burst release initially followed by a constant rate [53]. It was found that this NP has a greater in vitro cytotoxicity than the standard (adrenomycin) cell viability test provided the concentrations are the same for both. It was concluded that this greater cytotoxicity resulted because the cell absorbed this NP and curcumin was released near the cell membrane [53]. 

Another preclinical trial synthesized 131I-labelled multifunctional dendrimers/NPs for targeted tumor radiotherapy and single-photon emission computerized tomography (SPECT) imaging. These NPs were formed by conjugation of carboxylic acid functionalized methoxyl polyethylene glycol (mPEG-COOH), chlorotoxin conjugated polyethylene glycol (PEG-CTX), and 3-(4'-hydroxyphenyl) propionic acid-Osu (HPAO) onto amine-terminated Generation 5 polyamidoamine (G5 PAMAM) dendrimers. It was found that CTX-targeted multifunctional dendrimers had increased specificity for gliomas in comparison to other dendrimers. This specificity also allowed efficient SPECT imaging. The CTX-PEG was compared with the other two dendrimers, methoxypolyethylene glycol maleimide (PEG-MAL), and Na-131 to find out the radioactive inhibition of the tumor. The CTX-PEG dendrimer exhibited the highest tumor suppression in comparison to the other two [54].

Gold NPs were used in a preclinical trial to find out their neuro-protective effect on Parkinson’s disease. In this study PC12 cells were synthesized that are rat-derived cell lines and can differentiate into neuron-like cells. It was found that these gold NPs can prevent apoptosis of these PC12 cells thus halting the progression of Parkinson’s disease. The Nissl bodies of the mice on which the experiment was conducted, illustrated a therapeutic effect on nerve cell damage. There were no significant toxic effects found of these gold NPs on tissues on histological examinations of the heart, spleen, lung, kidneys, and brain. It was found that the highest concentration of these gold NPs was found in the spleen which is an indication of their effective clearance [55]. 

Pituitary acetylates cyclase-activating peptide (PACAP); a peptide that has a neuro-protective function was activated in mice by using gH26 liposomal NPs in a preclinical tryout. The study concluded that regular liposomes and liposomes conjugated with gH26 had no significant difference in the integrity of BBB however initially within 30 minutes and at higher concentrations of PACAP activating peptide gH26 liposomes were found to be more efficient in the transport of PACAP. Furthermore, it was found that gH26 liposomes had no effect on the integrity of BBB and hence had no toxic effects on the brain [56]. 

Clinical studies 

A phase 2 clinical trial for glioblastoma using pegylated liposomal doxorubicin for prolonged delivery of temozolomide and radiotherapy showed a 30.2% survival rate with a 12-month progression-free period. The toxicity of temozolomide with this NP was also found to be tolerable in patients. However, neither the administration of this NP nor the prolonged administration of temozolomide had a significant impact on the patient’s outcome [57]. 

Side effects, tolerability, and safety of gadolinium-based NPs, AGuIX in combination with standard complete brain radiotherapy was observed in a phase 1 clinical trial in patients with multiple brain metastases. The study concluded that these NPs are capable of accumulating in brain metastases thus increasing the effectiveness of radiotherapy [58]. This study entered a phase 2 clinical trial after these findings [59]. 

Future directions and recommendations

The future developments and potential risks of NP drug delivery for CNS pathologies are becoming increasingly important. Different factors such as size shape and surface properties play a critical role in achieving the best optimization for NPs. Altering of surface moieties (e.g. polyethylene glycol) and size can reduce the premature clearance of NPs which can lead to the accumulation of these agents in tumor cells/sites [60]. NPs smaller than 200 nm were found by different studies to be easily penetrating BBB, however, they should be larger than 5nm to avoid rapid renal clearance to be of optimal size [61-65]. Preferentially, the charge of NPs must be slightly cationic to avoid toxic effects while still allowing interaction with anionic BBB due to a slight cationic charge [65-68]. Antibody-conjugated NPs can enhance specific cellular uptake of NPs by receptor-mediated endocytosis [64,69]. 

Many targets are identified by NPs in CNS. Recent literature suggests that low density lipoprotein receptor-related protein 1 (LRP-1) is the target of NPs which is upregulated in brain tumors and is present in the capillary endothelium of BBB [70]. Target delivery by NPs can increase the availability of drugs to the brain but available literature suggests that more amounts of NPs are found to be accumulated in the liver than in the brain by employing targeted delivery of drugs through NPs [71-73]. 

Safety assessment of NPs is quite essential to consider before increasing its clinical use. Many factors such as the specific surface area of these particles can cause adverse effects such as the formation of reactive oxygen species [74]. However, it is considered that the administration of tetracycline for neurodegenerative diseases by NP drug delivery system has fewer side effects than conventional methods of tetracycline administration [75].

A clinical trial conducted on mice demonstrated that functionalized NPs that were combined with 1, 2-distearoyl-sn-glycerol-3-phosphoethanolamine-N-[amino(polyethylene glycol)-2000] (DSPE-PEG2K) could cross BBB easily in comparison to non-functionalized NPs or unconjugated NPs [76]. 

For the management of ischemic stroke, a clinical trial was performed by administering sequential therapies of NPs with anti-inflammatory and angiogenesis properties respectively by using a hydrogel to deliver vascular endothelial growth factor (VEGEF) and the ant-inflammatory drug 6-bromoindirubin-3′-oxime (BIO) for angiogenesis. The trial found that this sequential release of anti-inflammatory NPs followed by BIO NPs was more effective than administering both NPs separately [77]. The development of Multifunctional NPs is also a promising development for curing CNS diseases. Yang developed a multifunctional NP that can be used for imaging and simultaneously for delivery of therapeutic agents for treating brain tumors [78]. 

For regular use of NP drug delivery, a proper regulatory system for nanotechnology must be considered. Clinical and preclinical tryouts must be performed continuously to identify adverse effects and improve their efficacy. Quality control agencies must be employed for monitoring the production of nano-based drugs and robust quality control measures must be ensured. Along with these post-market surveillance team must also be recruited to ensure proper market handling of these products. 

## Conclusions

Liposomes, polymeric nanoparticles, dendrimers, and solid lipid nanoparticles are a few examples of the several types of nanoparticles that have been created and may be altered to improve targeting to certain brain regions, increase stability, and optimize drug release patterns. Opportunities in nanoparticle drug delivery for CNS disorders include leveraging the enhanced permeation and retention effect, employing active targeting strategies, achieving responsive drug release, developing multimodal imaging and therapeutic agents, and utilizing personalized medicine approaches. Several obstacles still exist in nanoparticle-based CNS medication delivery, despite recent advancements. It is necessary to undertake long-term safety evaluations, scale up production for clinical translation, and better understand how nanoparticles interact with the BBB. A rising understanding of CNS illnesses and continuous advances in nanotechnology, however, provide tremendous possibilities to enhance therapy results and address unmet healthcare needs.
